# Characterization of *Arabidopsis thaliana* Coq9 in the CoQ Biosynthetic Pathway

**DOI:** 10.3390/metabo13070813

**Published:** 2023-06-30

**Authors:** Mei Hu, Yan Jiang, Jing-Jing Xu

**Affiliations:** 1 Co-Innovation Center for Sustainable Forestry in Southern China, College of Biology and the Environment, Nanjing Forestry University, Nanjing 210037, China; 2 Shanghai Key Laboratory of Plant Functional Genomics and Resources, Shanghai Chenshan Botanical Garden, Shanghai 201602, China; 3 School of Life Sciences, Shanghai Normal University, Shanghai 200234, China

**Keywords:** coenzyme Q, plant metabolism, Coq9, *Arabidopsis*, mitochondria

## Abstract

Coenzyme Q, also known as ubiquinone, is a fat-soluble isoprene quinone that serves as a cofactor for numerous enzymes across all domains of life. However, the biosynthetic pathway for this important molecule in plants has been examined in only a limited number of studies. In yeast and mammals, Coq9, an isoprenoid-lipid-binding protein, is essential for CoQ biosynthesis. Previous studies showed that *Arabidopsis thaliana Coq9* failed to complement the fission yeast *Schizosaccharomyces pombe coq9* null mutant, and its function in plants remains unknown. In this study, we demonstrated that expression of *Arabidopsis Coq9* rescued the growth of a yeast temperature-sensitive *coq9* mutant and increased CoQ content. Phylogenetic analysis revealed that Coq9 is widely present in green plants. Green fluorescent protein (GFP) fusion experiments showed that *Arabidopsis* Coq9 is targeted to mitochondria. Disruption of the *Coq9* gene in *Arabidopsis* results in lower amounts of CoQ. Our work suggests that plant Coq9 is required for efficient CoQ biosynthesis. These findings provide new insights into the evolution of CoQ biosynthesis in plants. The identification of Coq9 as a key player in CoQ biosynthesis in plants opens up new avenues for understanding the regulation of this important metabolic pathway.

## 1. Introduction

Coenzyme Q (CoQ, ubiquinone) is a conserved redox-active, hydrophobic lipid that exists in most biological membranes. It serves as an electron transporter in the mitochondrial respiratory chain and accepts electrons from several other mitochondrial inner-membrane dehydrogenases [1]. Beyond its roles in mitochondria, the extramitochondrial CoQ has widespread cellular functions, including combatting ferroptosis and modulating membrane structure dynamics, as discussed in recent reviews [2].

CoQ is composed of a benzoquinone head group and a polyisoprenoid tail with a length that varies among species. The aromatic ring precursor and the polyprenyl-diphosphate tail precursor are synthesized in the cytosol and transported into the mitochondrial matrix. The yeast *Saccharomyces cerevisiae* has been used as an experimental model to study the eukaryotic CoQ biosynthesis pathway [3,4]. Currently, at least six enzymes (Coq1, Coq2, Coq3, Coq5, Coq6, and Coq7) and four auxiliary proteins (Coq4, Coq8, Coq9, and Coq11) are known to participate in CoQ biosynthesis in yeast mitochondria [2,3,5]. The genes responsible for CoQ biosynthesis in *S. cerevisiae* and humans are indicated in capital letters, while the corresponding proteins are denoted in uppercase letters for humans, with only the first letter capitalized in *S. cerevisiae*. In *Arabidopsis*, the genes and proteins are written with only the first letter capitalized, for instance, human gene *COQ9*, human protein COQ9, yeast gene *COQ9*, yeast protein Coq9, *Arabidopsis* gene *Coq9*, and *Arabidopsis* protein Coq9. The biosynthetic pathway of CoQ was thought to be highly conserved among species, but recent studies in plants have revealed substantial differences in the yeast pathway [6,7,8,9,10].

Recent studies have found that plants use a unique mitochondrial flavin-dependent monooxygenase enzyme (CoqF) to catalyze C6 hydroxylation of the aromatic ring [7,8]. By contrast, yeast and humans use an unrelated di-iron monooxygenase Coq7 [11]. The correct function of Coq7 requires the presence of Coq9, an isoprenoid-lipid-binding protein that forms a stable complex with Coq7 [12,13,14]. A central function of Coq9 is to enhance the substrate-binding capacity of Coq7, and its disruption results in accumulation of the Coq7 substrate (DMQ) [15,16]. Coq9 was originally detected in yeast genetic screens, and its deletion leads to the absence of CoQ [17]. He et al. [15] generated a *coq9* temperature-sensitive mutant using error-prone PCR, which had decreased CoQ content and grew poorly at the non-permissive temperature of 37 °C. Expression of human *COQ9* failed to complement yeast *coq9* null mutants but rescued the *coq9* temperature-sensitive mutant [18]. Previous studies have demonstrated that COQ9 is also required for CoQ biosynthesis in humans [19] and mice [20]. However, *Arabidopsis* Coq9 failed to complement the *S. pombe coq9* null mutant [21], and its function in planta has not been confirmed.

In this study, we tested whether plant Coq9 is involved in CoQ biosynthesis. We discovered that Coq9 is widely present in Viridiplantae, and expression of *Arabidopsis Coq9* rescued a yeast temperature-sensitive mutant. Finally, data from mutant analysis demonstrated that Coq9 is involved in the biosynthesis of CoQ in plants.

## 2. Materials and Methods

### 2.1. Plant Materials and Reagents

*Arabidopsis thaliana* ecotype Columbia-0 (Col-0) was used as the wild type. *Arabidopsis* plants were routinely grown in a greenhouse at 22 °C under a 16 h light/8 h dark cycle [22]. The seeds were surface-sterilized, placed on Murashige and Skoog medium, and incubated at 4 °C in the dark for 48 h. The plates were placed on the growth environment. Seven-day-old seedlings were transferred to soil. For experiments with *Nicotiana benthamiana*, plants were grown in a greenhouse at 25 °C under a 16 h light/8 h dark cycle. CoQ_4_, CoQ_6_, and CoQ_9_ were from Sigma-Aldrich, St. Louis, MO, USA).

### 2.2. Phylogenetic Analysis

Protein sequences of human COQ9 (NP_064708.1) and yeast Coq9 (QHB10353.1, strain CEN.PK113-7D) were obtained from NCBI. Protein sequences of Coq9 from *Botryococcus braunii*; *Sphagnum fallax*; *Physcomitrium patens*; *Ceratodon purpureus*; *Marchantia polymorpha*; *Selaginella moellendorffii*; *Ceratopteris richardii*; *Thuja plicata*; *Nymphaea colorata*; *Amborella trichopoda*; *Cinnamomum kanehirae*; *Dioscorea alata*; *Spirodela polyrhiza*; *Zostera marina*; *Panicum virgatum*; *Pharus latifolius*; *Oryza sativa*; *Brachypodium distachyon*; *Triticum aestivum*; *Hordeum vulgare*; *Sorghum bicolor*; *Eleusine coracana*; *Miscanthus sinensis*; *Chasmanthium laxum*; *Urochloa fusca*; *Setaria italica*; *Panicum hallii*; *Acorus americanus*; *Amaranthus hypochondriacus*; *Solanum lycopersicum*; *Vitis vinifera*; *Betula platyphylla*; *Cucumis sativus*; *Medicago truncatula*; *Glycine max*; *Aquilegia coerulea*; *Helianthus annuus*; *Anacardium occidentale*; *Prunus persica*; *Gossypium hirsutum*; *Manihot esculenta*; *Salix purpurea*; *Arabidopsis thaliana*; *Alyssum linifolium*; *Brassica rapa*; *Brassica oleracea*; *Crambe hispanica*; and *Sinapis alba* were obtained from Phytozome (https://phytozome-next.jgi.doe.gov/, accessed on 26 June 2023) using BLASTP with yeast Coq9 and human COQ9 as query sequences. The Coq9 sequences of *Coccomyxa subellipsoidea*; *Asparagus officinalis*; *Vanilla planifolia*; *Phalaenopsis equestris*; *Dendrobium catenatum*; *Musa acuminata*; *Zea mays*; *Coffea arabica*; *Nicotiana tabacum*; *Phaseolus vulgaris*; *Prunus mume*; *Prunus dulcis*; *Ricinus communis*; and *Populus trichocarpa* were obtained from NCBI using BLASTP with yeast Coq9 and human COQ9 as query sequences. All sequences were aligned with MAFFT [23] and trimmed by trimAl [24]. Furthermore, a phylogenetic tree was constructed using IQ-TREE [25]. ModelFinder [26] was used to determine the best model, and JTT + G4. Phylogenetic trees were visualized using iTOL [27].

### 2.3. Beta-Glucuronidase (GUS) Reporter Assay

Promoter regions of *AtCoq9* (2067 bp upstream of the start codon) were amplified by polymerase chain reaction (PCR) from *Arabidopsis* Col-0 genomic DNA with high-fidelity DNA polymerase (FastPfu, TransGen, Beijing, China) using the primers as listed (Supplementary Table S1). The amplified fragments were cloned into vector pDONR207 using Gateway technology. Then, the promoter region was introduced into the destination vector pGWB533. Afterwards, the clone was introduced into *Agrobacterium tumefaciens* strain GV3101 (purchased from WEIDI, Shanghai, China) and transformed into *Arabidopsis* through floral dipping [28].

GUS staining was performed according to [29]. Briefly, plant material was incubated in a staining buffer (0.5 mg/mL 5-bromo-4-chloro-3-indolyl-β-D-glucuronide in 0.1 M Na_2_HPO_4_, pH 7.0, 10 mM Na_2_EDTA, 0.5 mM K_3_Fe(CN)_6_, 0.5 mM K_4_Fe(CN)_6_, and 0.1% Triton X-100) at 37 °C in darkness overnight. The staining buffer was replaced by 70% ethanol to remove chlorophyll and visualized using a dissecting microscope (SZX7, OLYMPUS, Tokyo, Japan).

### 2.4. Subcellular Localization

Full-length coding regions of *AtCoq9* were amplified by PCR from *Arabidopsis* complementary DNAs (cDNAs) with high-fidelity DNA polymerase. Subsequently, the PCR product was cloned into the destination vector pGWB505 (a gift from Dr. Ping Xu). After sequence verification, the plasmid was transformed into *A. tumefaciens* strain GV3101 (pSoup-p19) (purchased from WEIDI).

Single colonies for each construct were resuspended in Yeast Extract Peptone medium (10 g/L yeast extract, 10 g/L Bacto peptone, and 5 g/L NaCl); supplied with 20 mg/L rifampicin, 40 mg/L gentamycin, and 75 mg/L spectinomycin; and cultured at 28 °C for 1 day. Bacterial cultures were centrifuged at 8000× *g* for 5 min. Cell pellets were washed with an induction buffer (10 mM MES, pH 5.7, 10 mM MgCl_2_, and 200 μM acetosyringone), and then resuspended in the induction buffer to a final OD_600_ of 0.3, which was determined using a NanoCrop 2000 spectrophotometer (Thermo Fisher Scientific, Waltham, MA, USA). Before injection, strains were incubated at room temperature for 3 h. The agrobacteria were then injected into the abaxial side of *N. benthamiana* leaves with a 1 mL needleless syringe. Vector CD3-991 (mitochondria targeted mCherry) was coinfiltrated with GFP constructs [30].

The transformed plants were grown in the greenhouse for 3 days. Images were captured using an Olympus FV10i confocal laser scanning microscope. Excitation wavelengths were 473 nm for GFP and 580 nm for mCherry. Emission fluorescence was captured by 490 to 540 nm for GFP and 610 nm for mCherry.

### 2.5. Quantitative Reverse-Transcription PCR

Tissue samples were collected and frozen in liquid nitrogen. Total RNA from 7-day-old seedlings, 14-day-old seedlings, 4-week-old rosette leaves, stems and stem leaves of 5-week-old plants, flowers, 3-day-old siliques, and 12-day-old siliques was isolated using TRIzol reagent (Thermo Fisher Scientific) according to the manufacturer’s protocol. Total RNA from mature seeds was isolated with the RNAprep Pure Plant Plus Kit (TIANGEN, Beijing, China). RNA was reverse-transcribed with the PrimeScript RT reagent Kit with the gDNA Eraser (Takara, Dalian, China). qRT-PCR was performed in triplicate on Mastercycler ep Realplex2 (Eppendorf, Hamburg, Germany), using TB Green Premix Ex Taq (Takara). PCR cycling was performed as follows: 30 s at 95 °C, followed by 40 cycles of 10 s at 95 °C, 15 s at 60 °C, and 20 s at 72 °C. To confirm the specificity of primer amplification, a melting curve was generated by gradually increasing the temperature from 75 °C to 95 °C in increments of 0.5 °C. Three technical replicates were performed for each tissue sample. The 2^−ΔΔCt^ method was used to determine the relative expression. The *Arabidopsis* gene *PP2AA3* (At1g13320) was used as an endogenous control [31]. The primers for qRT-PCR analysis are presented in Supplementary Table S1.

### 2.6. Yeast Strain and Complementation Assays

*Saccharomyces cerevisiae* strain BY4742 was used in this study as control strain. Δ*coq9* (Y14150) was purchased from Euroscarf. Yeast was transformed using the Frozen-EZ Yeast Transformation kit (ZYMO, Irvine, CA, USA) according to the manufacturer’s protocol and grown at 30 °C on SD-Leu-His agar media (0.67% BD Difco yeast nitrogen base with ammonium sulfate, 0.62 g/L Clontech Dropout supplement -His/-Leu/-Trp, 20 mg/L tryptophan, 2% dextrose and 2% Bacto agar).

The yeast *COQ9* gene was amplified from the genomic DNA of BY4742 by PCR. The ClonExpress II One Step Cloning Kit (Vazyme Biotech, Nanjing, China) was used to insert PCR amplicons into the XbaI- and BamHI-linearized pRS315 vector. The plasmid TS19 was generated according to [15]. Site-directed mutagenesis (E55G, R107G, and Q256L) was performed using the fully assembled ScCoq9-pRS315 plasmid as a template, with specific mutations generated by PCR. Full-length coding regions of *AtCoq9* were amplified by PCR from *Arabidopsis* cDNAs and cloned into pRS423 controlled by the *S. cerevisiae COQ8* promoter and terminator. Human *COQ9* gene sequence was synthesized (GenScript) and cloned into pRS423. The mitochondrial targeting sequence of yeast Coq3 was fused with AtCoq9 and human COQ9.

For complementation analyses, serial dilutions of yeast cells were dropped onto SD-Leu-His agar media or YPG (3% glycerol, 1% yeast extract, 2% peptone, and 2% Bacto agar) plates.

### 2.7. Generation of coq9 Mutants Using CRISPR-Cas9

To design the gRNA, we used the web tools CRISPR-PLANT (http://omap.org/crispr/CRISPRsearch.html, accessed on 26 June 2023) and selected one target sequence (GCGAACGATGTACCGAACGG) in the first exon of *AtCoq9*. Golden Gate cloning [32,33] was used to construct the gRNA in the level 1 vector. Level 1 constructs pICSL11059::35S::hptII, pICSL11049::AtUbi10::Cas9, pICH47751:: AtU6-26p::sgRNA1, and the linker pICH41766 were assembled into the level 2 vector pICSL4723. The MoClo Toolkit (Addgene kit #1000000044) and pICSL11059 (Addgene plasmid #68263) were obtained from Addgene. The final clone was introduced into *A. tumefaciens* strain GV3101 and transformed into *Arabidopsis* through floral dipping [28]. Genome sequences of *AtCoq9* in T1 lines were amplified and sequenced.

### 2.8. Analysis of CoQ Contents

Yeast cells were grown at 25 °C or 30 °C on SD-Leu-His liquid medium with 2% glucose until OD_600_ reached ~1. Cells with a quantity totaling 10 mL were centrifuged, and the pellet mass was determined. Glass beads (0.5 mm diameter, 100 μL), 1 mL of isopropanol, and internal standard CoQ_4_ (0.1 mg/L) were added to the cell pellets (50 mg wet weight). Samples were vortexed (2 min) and extracted in a sonicator bath for 30 min. After centrifugation at 12,000 rpm for 10 min, the pellets were extracted with 1 mL of isopropanol in a sonicator bath for 30 min and centrifuged at 12,000 rpm for 10 min. The supernatant from two extractions were combined for LC-MS analysis.

For *Arabidopsis*, samples were harvested, frozen in liquid nitrogen, and freeze-dried. Ten milligrams of freeze-dried samples were extracted with 1 mL of isopropanol in a sonicator bath for 60 min.

LC-MS/MS analyses were carried out using an Agilent 1260 high-performance liquid chromatography (HPLC) and 6460 Triple Quadrupole LC-MS system. Multiple reaction monitoring in positive ion mode was used with the following parameters: gas temperature, 300 °C; gas flow rate, 11 L/min; and nebulizer, 35 psi. The sample injection volume was 5 μL. An AgilentXDB-C18 column (2.1 × 50 mm, 3.5 μm particles) was used for reverse-phase chromatography with mobile phases of A (isopropanol) and B (acetonitrile/H_2_O, 7:1, 10 mM ammonium acetate). The following gradient was used for separation with a flow rate of 0.4 mL/min: 0–6 min (35–85% A), 6–6.5 min (85–35% A), and 6.5–10 min (35% A). CoQ_4_ was measured using monitored transitions with the following parameters: precursor ion, 455.3; product ion, 197.1; dwell, 100 ms; fragmentor voltage, 110 V; collision energy, 16 V; and cell accelerator voltage, 4 V. CoQ_6_ was measured using monitored transitions with the following parameters: precursor ion, 591.4; product ion, 197.1; dwell, 100 ms; fragmentor voltage, 182 V; collision energy, 28 V; and cell accelerator voltage, 4 V. CoQ_9_ was measured using monitored transitions with the following parameters: precursor ion, 795.6; product ion, 197.1; dwell, 100 ms; fragmentor voltage, 216 V; collision energy, 36 V; and cell accelerator voltage, 4 V.

## 3. Results

### 3.1. Phylogenetic Analysis of Plant Coq9

**Figure 1:**
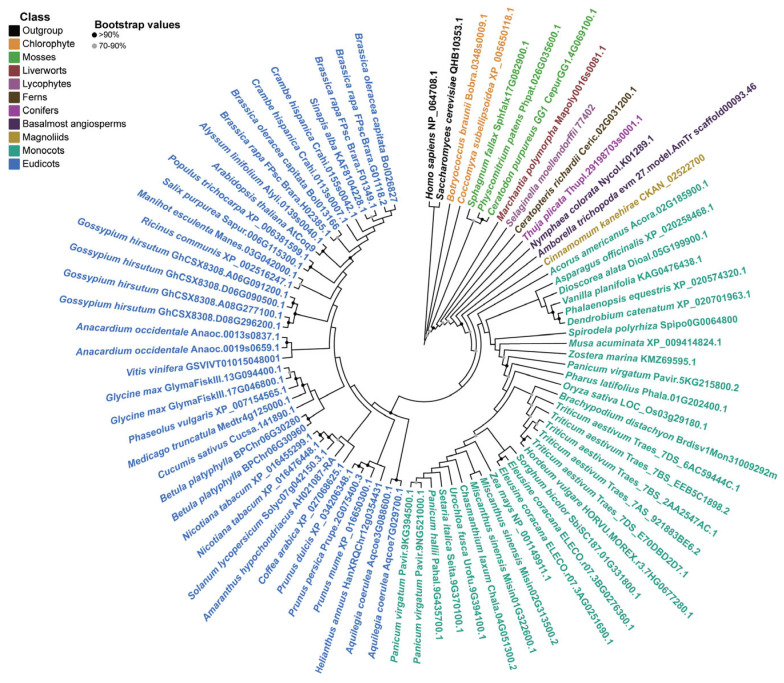
Coq9 is widely distributed in green plants. Amino acid sequences from two chlorophytes, three mosses, one liverwort, one lycophyte, one fern, one conifer, and fifty-three angiosperms were aligned using MAFFT. Maximum-likelihood phylogenetic tree was constructed using IQ-TREE under the JTT + G4 model selected by ModelFinder. The tree was rooted using human COQ9 (NP_064708.1) and yeast Coq9 (QHB10353.1) as an outgroup. Black circles are used to represent bootstrap values over 90%, and grey circles are used represent bootstrap values between 70% and 90%.

To examine the distribution of Coq9 in green plants, we screened the genomes of 62 representative species of Viridiplantae via BLAST, using yeast Coq9 and human COQ9 as query sequences. A phylogenetic tree based on sequences from two chlorophytes, three mosses, one liverwort, one lycophyte, one fern, one conifer, and fifty-three angiosperms was inferred using the maximum-likelihood method (Figure 1). To root the tree, human COQ9 and yeast Coq9 were included as an outgroup. Coq9 was identified in all taxa investigated. The phylogenetic tree of Coq9s complies with the branches of Viridiplantae.

Further analysis of the numbers of *Coq9* genes showed that diploid species, such as *Arabidopsis*, *Solanum lycopersicum*, *Oryza sativa*, *Zea mays*, and *Sorghum bicolor*, retained one copy, the palaeopolyploid *Glycine max* contained two copies, and the hexaploid *Triticum aestivum* had three copies. These data suggest that Coq9 was widely present in Viridiplantae and did not undergo multiple duplications.

### 3.2. Expression Patterns of AtCoq9

**Figure 2:**
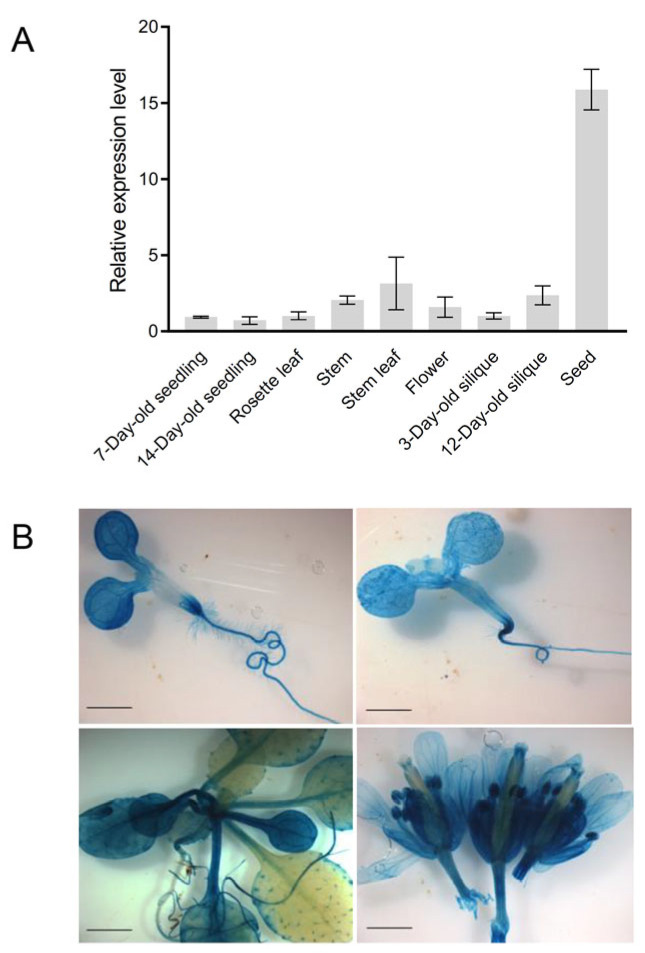
Expression patterns of *AtCoq9*. (**A**) qRT-PCR analysis of *AtCoq9* expression levels in different organs. *PP2AA3* was used as the internal reference. Data are means of three biological replicates ±SE. (**B**) Histochemical GUS staining of *ProAtCoq9::GUS* transgenic plants. Shown are 7-day-old seedling, 14-day-old seedling, four-week-old plant, and inflorescence. Scale bars, 2 mm.

To profile the expression patterns of *AtCoq9*, quantitative reverse-transcription PCR (qRT-PCR) was performed using *Arabidopsis* RNAs from the seedlings, rosettes, stems, stem leaves, flowers, siliques, and mature seeds. The *AtCoq9* gene was widely expressed, with the highest levels in mature seeds (Figure 2A), where the CoQ contents were also most abundant [7]. To further examine the spatiotemporal expression of *AtCoq9*, transgenic *Arabidopsis* plants carrying a *ProAtcoq9::GUS* construct were generated. Three independent transgenic lines were selected for GUS staining (Figure 2B). Ubiquitous GUS activity was observed in 7-day-old seedlings and 14-day-old seedlings. In four-week-old plants, a weak signal could be observed in young leaves, while strong expression was found in old leaves. In inflorescence, *AtCoq9* was again widely expressed. In summary, *AtCoq9* is ubiquitously expressed throughout plant development.

### 3.3. AtCoq9 Is Localized in Mitochondria

**Figure 3:**
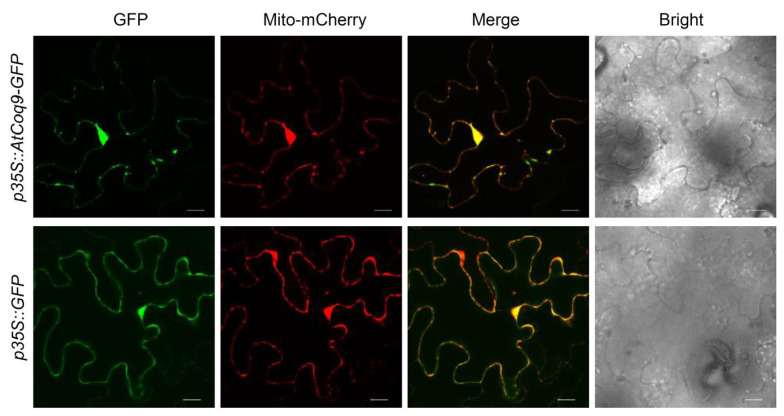
AtCoq9 is localized in mitochondria. GFP was fused to the C-terminus of AtCoq9 and transiently expressed in *Nicotiana benthamiana* with the mCherry-fused mitochondrial marker (CD3-991). Shown are confocal fluorescence micrographs of epidermal cells, images of the emission of GFP, mCherry, and the merged images. Scale bars, 20 μm.

AtCoq9 contains the N-terminal mitochondrial targeting sequence, as predicted by TargetP [34]. To examine the subcellular localization of AtCoq9, green fluorescent protein (GFP) was fused to the carboxy terminus of AtCoq9 and transiently expressed in *N. benthamiana* leaves, along with the mitochondrial mCherry-tagged marker (Figure 3). The signal of GFP fused with AtCoq9 did overlap with the fluorescence of the mCherry mitochondrial marker, thus confirming its predicted mitochondrial localization, which is consistent with previous high-throughput studies [35].

### 3.4. AtCoq9 Rescues the Yeast coq9 Point Mutant

Previous studies showed that expression of either *AtCoq9* or human *COQ9* did not complement the *S. pombe coq9* null mutant [21]. Meanwhile, expression of human *COQ9* rescued the *S. cerevisiae* temperature-sensitive mutant *coq9-ts19*, which requires the presence of E55G, R107G, and Q256L mutations [18]. Thus, we tested whether expression of *AtCoq9* could also rescues a *coq9* temperature-sensitive mutant. We transformed the TS19 plasmids containing the yeast *COQ9* gene with three mutations into the yeast *coq9* null mutant to generate a *coq9* temperature-sensitive mutant according to [15]. The *coq9-ts19* mutant had decreased CoQ_6_ content and failed to grow at the non-permissive temperature of 37 °C [15].

**Figure 4:**
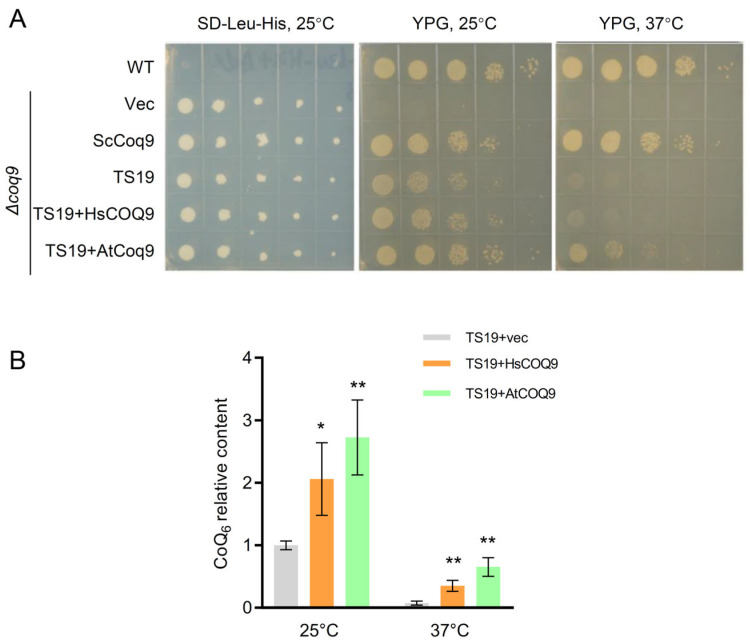
AtCoq9 rescues a yeast *coq9* temperature-sensitive mutant. (**A**) Growth of *S. cerevisiae* strains transformed with the following plasmids: WT strain BY4742 transformed with the empty vectors or the *coq9* mutant strain transformed with the empty vectors (vec), empty vector pRS315 and yeast *COQ9* on pRS423 (ScCoq9), empty vector pRS423 and TS19 on pRS315 (TS19), TS19 on pRS315 and human *COQ9* on pRS423 (TS19 + HsCOQ9), and TS19 on pRS315 and pRS423-*AtCoq9* (TS19 + AtCoq9). Serial dilutions were spotted onto SD-Leu-His or YPG plate media and incubated at either 25 °C or 37 °C. (**B**) Quantification of CoQ_6_ contents. Yeast cells were cultivated in SD-Leu-His liquid media containing 2% glucose and grown overnight at either 25 °C or 37 °C. Lipids were extracted and analyzed by LC-MS. Asterisks indicate significant difference compared to TS19 + vec with * *p* < 0.05 and ** *p* < 0.01 (two-tailed Student’s *t* tests). Data are means of three biological replicates ± SE.

Expression of *AtCoq9* containing the yeast Coq3 mitochondrial targeting sequence restored the growth of the *coq9-ts19* mutant at 37 °C on SD-Leu-His media (Figure 4A). Analysis of the lipid extract by LC-MS/MS indicated that the presence of AtCoq9 or human COQ9 significantly increased the amount of CoQ_6_ at both permissive (25 °C) and non-permissive temperatures (37 °C), with AtCoq9 providing noticeably more rescue (Figure 4B). These results indicate a functional conservation of Coq9 across different organisms.

### 3.5. AtCoq9 Defective Mutants Contained Less CoQ

**Figure 5:**
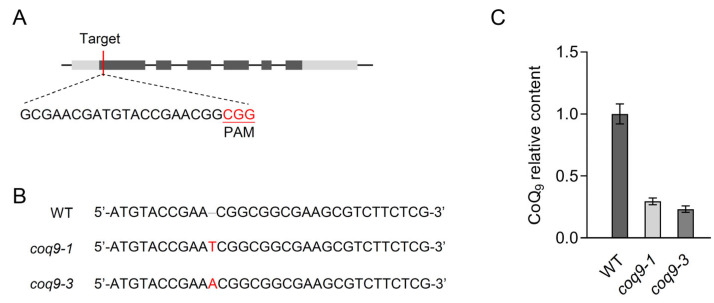
Coq9 is required for efficient CoQ biosynthesis. (**A**) Graphical representation of *AtCoq9* gene and position of gRNA. Exons are indicated by gray boxes. The CRISPR-Cas9-targeted sequence is shown in black, and the protospacer-adjacent motif (PAM) sequence is shown in red. (**B**) DNA sequencing indicates the 1 bp insertion (red) in the obtained *coq9-1* and *coq9-3* mutants. (**C**) Relative content of CoQ_9_ in extracts of rosettes in WT, *coq9-1*, and *coq9-3*. Data are means of four to five biological replicates ± SE.

To study the in vivo function of AtCoq9, we generated *AtCoq9*-knockout mutants by CRISPR-Cas9-mediated genome editing.gRNA was designed to target the first exon of *AtCoq9* (Figure 5A), and the construct carrying the gRNA and Cas9 protein was transformed in *Arabidopsis* plants. Two independent knockout alleles were identified in the T1 generation, both of which contained 1 bp insertion and resulted in premature stop codons for *AtCoq9* (Figure 5B). Homozygous-knockout alleles were obtained in the T2 generation and used for further analyses. In *coq9-1* and *coq9-3* mutants, the CoQ_9_ levels were reduced by 70 to 80% compared to those of wild-type plants (Figure 5C). However, no phenotypic difference was observed between *coq9* mutants and the wild type. Together, these results demonstrate that AtCoq9 is involved in the biosynthesis of CoQ.

## 4. Discussion

CoQ is an isoprenoid quinone present in all eukaryotes. The biosynthetic pathway of CoQ in plants has not been fully defined to date [6]. In this study, we found that expression of *Arabidopsis Coq9* rescues the yeast *coq9* temperature-sensitive mutant. The knockout mutants of *Arabidopsis Coq9* contain less CoQ. These findings suggest the evolutionary conservation of Coq9 between plants, yeast, and humans. It also demonstrates that Coq9 is required for efficient CoQ biosynthesis in *Arabidopsis*.

CoQ is essential for embryogenesis in *Arabidopsis*. Knockout mutants lacking some of the *Coq* genes, such as *Coq1* [36], *Coq2* [37], *Coq3* [38], or *CoqF* [7], are embryo-lethal, and no homozygous mutant plants could be recovered from seeds of heterozygous plants after self-pollination. However, homozygous plants of *coq9* knockout mutants were identified, suggesting that Coq9 is not essential for embryo development. In addition, we found that the *coq9* knockout mutants still produced small amount of CoQ. The data consistent with the idea that the considerable reduction in CoQ levels in *Arabidopsis* mutants still enables normal embryo development. Currently, there is relatively little research on the relationship between CoQ metabolism and plant growth and development, and it is worth further study in the future.

In yeast, deletion of the *COQ9* gene results in a complete loss of CoQ synthesis and consequently a failure to grow on a nonfermentable carbon source [17]. However, unlike yeast, *Arabidopsis* lacking *Coq9* is not completely CoQ deficient. Although *Arabidopsis* Coq9 rescued the yeast *coq9* temperature-sensitive mutant, it failed to complement the *S. pombe coq9* null mutant [21]. Therefore, it is likely that the function of Coq9 may not be fully conserved between yeast and *Arabidopsis*.

In yeast and mammals, Coq9 is required for the function of Coq7, a carboxylate-bridged diiron hydroxylase responsible for catalyzing the penultimate step in CoQ biosynthesis. The position of the Coq9 protein in the CoQ biosynthesis pathway has been reviewed recently [2,5]. However, plants lack Coq7, and C6 hydroxylation is catalyzed by CoqF, a flavin-dependent monooxygenase [7,8]. The functional relationship between Coq9 and CoqF in plants is not yet clear. Moreover, Coq9 has been shown to be present in alpha proteobacterial organisms that do not have Coq7 and has been considered to be an ancestral protein of the CoQ biosynthetic pathway [39]. We speculate that Coq9 may perform other roles in addition to assisting the hydroxylase reaction of Coq7. It is interesting to investigate the precise biochemical functions of Coq9 in CoQ biosynthesis in species without Coq7.

In conclusion, this work reveals that Coq9 plays an important role in CoQ biosynthesis in *Arabidopsis*. It also describes the functional conservation of Coq9 across different organisms. Our findings add to the understanding of the CoQ biosynthetic pathway in plants and provide valuable insights for further exploration of the roles of Coq9. Further studies are needed to elucidate the precise mechanism of Coq9 function in CoQ biosynthesis in plants, as well as to explore the potential applications of CoQ in agriculture and medicine.

## Data Availability

Primary data are available in Supplementary Table S1.

## Supplementary Materials

The following supporting information can be downloaded at: https://www.mdpi.com/article/10.3390/metabo13070813/s1, Table S1: Primers used in this study.
