# Supplementary material for: Characterization of Arabidopsis thaliana Coq9 in the CoQ Biosynthetic Pathway
Source: Metabolites. 2023 Jun 30;13(7):813. doi: 10.3390/metabo13070813 (PMC10385794; doi:10.3390/metabo13070813)
Supplement: Supplementary file 1 [file metabolites-13-00813-s001.zip › metabolites-2473427-supplementary.pdf]

Supplemental Table S1 Primers used in this study.

Primer Name	Sequence (5' > 3')
**qRT-PCR analysis**	
PP2A-RT-s	ACTCCTCTGGCTAAGCGACT
PP2A-RT-a	CGCACCATTGGCATGTCATC
AtCoq9-RT-s	AAGCGATGATGGCTGGTTCA
AtCoq9-RT-a	AGAGCTTGGGGCCATTTTGA
**Promoter gateway cloning**	
AtCoq9-pro-F	GGGGACAAGTTTGTACAAAAAAGCAGGCTTCTTGC TACACAAGCTACAAGC
AtCoq9-pro-R	GGGGACCACTTTGTACAAGAAAGCTGGGTTCATCG TTCGCCGGAGTAAG
**CDS gateway cloning**	
AtCoq9-pDonr-F	GGGGACAAGTTTGTACAAAAAAGCAGGCTTC ATGTACCGAACGGCGGCG
AtCoq9-pDoner-R	GGGGACCACTTTGTACAAGAAAGCTGGGTT AAACGCAGAGCCACCACT
**Primers used for generating TS19**	
ScCoq9-pRS315-F	GGTGGCGGCCGCTCTAGACCGGGTTCAGAGGTAAA AGG
ScCoq9-pRS315-R	GCAGCCCGGGGGATCCGGGACAAGCAGGAAGAAC TA
ScCoq9-E55G-f	GCTCTACAAAAGTTTGTTCCCGGGCACGGTTTC
ScCoq9-E55G-r	GAAACCGTGCCCGGGAACAAACTTTTGTAGAGC
ScCoq9-R107G-f	GTTTCAATTGGTAGATAAAGGGTATCGTTTAAC
ScCoq9-R107G-r	GTTAAACGATACCCTTTATCTACCAATTGAAAC
ScCoq9-Q256L-f	CTACGGTAAATTTAATCAAATCTCTATTAGTTA
ScCoq9-Q256L-r	TAACTAATAGAGATTTGATTAAATTTACCGTAG
**Primers used for complementation assays**	
ScCoq8-pro-F	TCCACCGCGGTGGCGGCCGCGATCCGGGTGTTCGG A
ScCoq8-pro-R	CTTAACAACATGGGGGATCCCATATCGAACGATATC T
ScCoq8-ter-F	AATACTTCCCCGCTATTTG
ScCoq8-ter-R	TACCGGGCCCCCCCTCGAGCTATTGGCAGAAGGATT
ScCoq3-MTS-F	GGGATCCCCCATGTTGTTAAGATCTAGA
ScCoq3-MTS-R	TGTGCTCTTACATCTCGT
AtCoq9-PRS423-s	ACGAGATGTAAGAGCACAATGTACCGAACGGCGGC G
AtCoq9-PRS423-a	AATAGCGGGGAAGTATTTCAAAACGCAGAGCCACC
HsCOQ9-PRS423-s	CGAGATGTAAGAGCACA ATGGCGGCGGCGGCGGTAT
HsCOQ9-PRS423-a	AATAGCGGGGAAGTATTTCACCGACGCTGGTTTAG

**Primers used for generating CRISPR plasmids**	
gRNA-F	TGTGGTCTCAATTG GCGAACGATGTACCGAACGG GTTTTAGAGCTAGAAATAGCAAG
gRNA-R	TGTGGTCTCAAGCGTAATGCCAACTTTGTAC
